# The spectrum of coincident entities with small lymphocytic lymphoma/chronic lymphocytic leukemia (SLL/CLL) diagnosed by cytology

**DOI:** 10.4103/1742-6413.70966

**Published:** 2010-10-11

**Authors:** Hannah A. Kastenbaum, Walid E. Khalbuss, Raymond E. Felgar, Ronald Stoller, Sara E. Monaco

**Affiliations:** Department of Pathology, University of Pittsburgh Medical Center, Pittsburgh, Pennsylvania, USA; 1 Department of Medicine, University of Pittsburgh Medical Center, Pittsburgh, Pennsylvania, USA

**Keywords:** Chronic lymphocytic leukemia, cytopathology, SLL/CLL, small lymphocytic lymphoma

## Abstract

**Background:**

The cytologic diagnosis of Small lymphocytic lymphoma/chronic lymphocytic leukemia (SLL/CLL) often relies on finding a small lymphoid population with the characteristic immunoprofile by ancillary testing. There are only a few reports of other processes identified with SLL/CLL. The aim of this study was to review the fine needle aspiration (FNA) and touch prep (TP) diagnoses of SLL/CLL in order to identify any coincident entities.

**Materials and Methods:**

We retrospectively reviewed all FNA and TP cytology cases between January 2005 and May 2009 with a diagnosis of SLL/CLL to determine the presence of any coincident process.

**Results:**

We identified 29 cases, including 23 FNAs and six TPs, from 23 patients. Ancillary studies were utilized in 97% of the cases, including flow cytometry (FC, 79%), immunohistochemistry (IHC, 55%), fluorescent *in situ* hybridization studies (24%) and special stains (7%). Coincident entities were identified in nine cases (31%) and included seven (28%) neoplastic entities (Hodgkin lymphoma [HL], adenocarcinoma, squamous cell carcinoma, seminoma) and two (7%) non-neoplastic entities (infection and immunoglobulin containing cells). Six cases (21%) suspicious for large cell transformation were also identified.

**Conclusion:**

In our review of SLL/CLL, coincident entities were present in 31% of the cases and included a spectrum of non-neoplastic and neoplastic processes. FC was the most frequently utilized ancillary test, but IHC provided important information by excluding a mantle cell lymphoma or confirming a coincident process. Thus, cytomorphologic evaluation in these patients is important due to the high risk of a coincident process that may not be apparent by FC alone and may require clinical management.

## INTRODUCTION

Small lymphocytic lymphoma/chronic lymphocytic leukemia (SLL/CLL) is a low-grade non-Hodgkin Bcell lymphoma (NHL). The cytological diagnosis of SLL/CLL often relies on finding a homogenous small lymphoid population and establishing the characteristic immunoprofile via ancillary studies. As with most lymphomas diagnosed by fine needle aspiration (FNA), ancillary studies have an important role is establishing the diagnosis of lymphoma and in helping to subtype the lymphoma.[1–3]

Because SLL/CLL is an indolent lymphoma, patients can live for many years after a diagnosis and usually do not require aggressive treatment.[4] In fact, patients with asymptomatic, early-stage disease can be monitored without therapy.[5] Patients with SLL/CLL often present with persistent or recurrent lymphadenopathy; however, patients can also present asymptomatically and have SLL/CLL discovered incidentally upon lymph node evaluation or routine complete blood count. Furthermore, given the slow progression of the disease, the patients will often succumb to additional medical problems.[4]

The occurrence of secondary malignancies arising in SLL/CLL is well known and, according to the National Cancer Institute’s Surveillance, Epidemiology, and End Results (SEER) Program, there is an overall incidence of second cancers in SLL/CLL patients of about 8.7%.[6] In the literature, there are reports of other hematolymphoid malignancies identified with SLL/CLL, including transformation to a more aggressive lymphoma, such as a large cell NHL or Hodgkin lymphoma (HL).[6–9] There are also reports of patients with SLL/CLL having simultaneous T-cell lymphoma[8,10,11] or multiple myeloma.[12–14] In addition, simultaneous non-lymphoid malignancies have been reported in SLL/CLL, including squamous cell carcinomas,[15–21] Merkel cell carcinoma[22] and other malignancies.[6]

The transformation of SLL/CLL to a high-grade lymphoma, or Richter syndrome (RS), is well described, with a reported incidence of 2.2–10%,[23–26] and a greater risk has been reported in young patients diagnosed with SLL/CLL.[24] Classically, RS consists of a large cell NHL, which is most often a diffuse large B-cell lymphoma. However, SLL/CLL with HL transformation has also been described, with a reported incidence of 1% or less,[6,26,27] and is considered one of the most common secondary malignancies arising in these patients.[6]

In the cytology literature, there are only a few case reports and rare case series of simultaneous entities seen with SLL/CLL;[9,15,16,18,21,28,29] however, no studies have described the full spectrum of cytomorphologic lesions seen in these patients. The aim of this study was to review the FNA biopsy and touch prep (TP) diagnoses of SLL/CLL at our institution in order to identify any coincident diagnoses in the background of the lymphoma, to correlate the cytology with available pathologic follow-up and to look at the ancillary studies used.

## MATERIALS AND METHODS

We searched the pathology database at our institution for all FNA and TP cytology cases between January 2005 and May 2009 with a diagnosis of SLL/CLL. All exfoliative cytology cases were excluded. The slides from each case were retrospectively reviewed to confirm the diagnosis rendered. The study was reviewed and approved by the institutional review board of the University of Pittsburgh Medical Center (IRB# PRO09040344).

FNAs were performed by a cytopathologist using palpation or a radiologist/surgeon using image guidance. Most cases used either 23- or 25-gauge needles and had on-site immediate evaluation by a cytopathologist for adequacy assessment. On-site evaluation involved examination of a Diff-Quik™ (Protocol Hema 3; Fisher Scientific, Kalamazoo, MI, USA)-stained smear from each pass to confirm adequate sampling of the lesion and to issue a preliminary interpretation to the clinician. Additional smears were fixed in 95% alcohol. Air-dried smears on charged slides were prepared for potential fluorescent *in situ* hybridization (FISH) studies. Needle rinses and/or additional passes were rinsed in formalin for cell block preparation or in a balanced salt solution (Invitrogen, Life Technologies, Grand Island, NY, USA) for flow cytometry. The total number of passes varied based on the ability to obtain adequate material and the ability of the procedure to be tolerated but, in general, three to five passes were used.

At the time of final interpretation, the Papanicolaou and Diff-Quik™-stained smears, in addition to the hematoxylin and eosin-stained sections of the cell block, were evaluated and interpreted in conjunction with the results from ancillary studies to render a final diagnosis.

When retrospectively reviewed, the following data were gathered from each case: the cytologic diagnosis, clinical history, age, gender, site, use of image-guidance, ancillary studies utilized, presence of coincident processes and histological follow-up, if available.

Flow cytometry (FC) studies were performed on the cell suspensions in RPMI. The interpretation of the FC data was performed by one of eight hematopathologists with American Board of Pathology and Hematology certification or equivalent qualifications, who generated reports. The FC reports were then available for the cytopathologist to correlate with the cytomorphology and other findings. Immunohistochemistry (IHC) and special stains were performed on deparaffinized, formalin-fixed sections from the cell block using a variety of antibodies on the Ventana Benchmark XT system (Ventana Medical Systems, Tucson, AZ, USA) or special stains in the histology laboratory, respectively. IHC was ordered in an effort to further characterize the cells present (epithelial, hematolymphoid, mesenchymal, etc), to determine the immunoprofile of particular cells, and commonly, to exclude the possibility of a mantle cell lymphoma (with a cyclin D1 stain). FISH studies were performed on direct unstained smears on charged slides or cell blocks for various gene rearrangements and/or other specific chromosomal translocations. FISH studies were primarily carried out using probes for the *IGH* gene rearrangement or dual-color dual-fusion probes for characteristic translocations, as previously described.[3]

In a majority of the cases, the diagnosis of SLL/CLL was based on finding a monoclonal B-cell population by FC, expressing CD19, CD5 and CD23 and lacking CD10 and FMC-7. In positive FC cases, ZAP-70 and CD38 were also performed if enough cells were available. The majority of these cases also had cyclinD1 performed on cell block or histologic material to exclude the possibility of a mantle cell lymphoma. When FC was not available, the diagnosis was based on the morphology of the lymph nodes, clinical history, peripheral blood findings and, in some cases, IHC results to confirm a CD5-positive B-cell population lacking cyclin D1. A transformation to HL was diagnosed in cases with characteristic Reed-Sternberg cells identified cytomorphologically, along with immunohistochemical positivity for CD15/LeuM1 and CD30, and a polymorphous background of HL with neoplastic B-cells characteristic of SLL/CLL by FC or IHC, as used in other studies.[9] A diagnosis of “suspicious for a large cell transformation” was generally made in cases with scattered atypical cells (approximately >10%), showing intermediate-to-large-sized nuclei, prominent nucleoli, a moderate amount of basophilic cytoplasm and essentially falling short of being diagnostic of a prolymphocytic leukemia, large cell NHL or HL. Although this cutoff of 10% may be low, we have chosen a more conservative approach in an effort to recommend follow-up biopsies in patients who potentially could have CLL/PL (increased prolymphocytes, >10% prolymphocytes), transformation to HL or a transformation to a large cell NHL arising in CLL/SLL.

## RESULTS

### Clinicopathologic characteristics

We identified 23 FNA and six TP specimens with a diagnosis of SLL/CLL from 23 patients, including 15 males and eight females [Table 1]. The patients ranged in age from 45 to 86 years (mean, 69 years). The sites of the specimens included 25 lymph nodes, three major salivary glands and one lung mass. Twenty-three (79%) patients had a prior history of SLL/CLL. The FNA specimens (23, 79%) were acquired via pathologist-performed FNA (17 cases, 74%) or image-guided FNA (six cases, 26%). Six cases (21%) were acquired via intraoperative TP examination. Table 2 shows the cases with diagnoses and follow-up [Table 2].

**Table 1:** Clinicopathologic findings in 29 FNA and TP diagnoses of SLL/CLL

	*Number*	%
Total cases	29	
#patients	23	
Average age (years)	69	
Male	15	65
Female	8	35
Primary diagnosis of SLL/CLL	6	21
Secondary diagnosis of SLL/CLL	23	79
Touch preparations	6	21
Fine needle aspiration (FNA)	23	79
By pathologist	17	74
USG FNA	3	13
EBUS FNA	2	9
CTG FNA	1	4
Location		
Head and neck	13	45
Mediastinal/lung	7	24
Axillary	6	21
Other	3	10
Tissue type		
Lymph node	25	86
Salivary gland	3	10
Lung	1	4
Pathologic follow-up	21	72
Ancillary studies performed	28	97
Flow cytometry	23	79
IHC	16	55
FISH	7	24
Special stains	2	7

**Table 2:** Cases of SLL/CLL with cytology and follow-up

*Case*	*Specimen type*	*Anatomic site*	*Cytology*	*Pathologic follow-up*
1	FNA-P	LN	SLL/CLL	BM biopsy, PB and surgical biopsy (+SLL/CLL)
2	FNA-P	LN	SLL/CLL and Hodgkin lymphoma	Surgical biopsy (+SLL/CLL and Hodgkin lymphoma)
3	USG-FNA	LN	SLL/CLL, suspicious for transformation	None
4	FNA-P	LN	SLL/CLL	PB (+SLL/CLL)
5	FNA-P	LN	SLL/CLL	None
6	FNA-P	LN	SLL/CLL	BM biopsy (+SLL/CLL)
7	FNA-P	LN	SLL/CLL, suspicious for transformation	BM biopsy (+SLL/CLL with possible incipient transformation)
8	FNA-P	LN	SLL/CLL, suspicious for transformation	BM biopsy (+SLL/CLL, no transformation)
9	FNA-P	LN	SLL/CLL, suspicious for transformation	FNA (see below)
10	FNA-P	LN	SLL/CLL, suspicious for transformation	FNA (see below)
11	FNA-P	LN	SLL/CLL and Hodgkin lymphoma	None
12	EBUS-FNA	LN	SLL/CLL	PB (+SLL/CLL)
13	CTG-FNA	LN	SLL/CLL	FNA (see below)
14	FNA-P	LN	SLL/CLL	Autopsy (+SLL/CLL)
15	FNA-P	Salivary gland	SLL/CLL	None
16	FNA-P	LN	SLL/CLL and Hodgkin lymphoma	None
17	FNA-P	LN	SLL/CLL and SqCC	Surgical excision (+SqCC and SLL/CLL)
18	TP	Salivary gland	SLL/CLL	BM biopsy and concurrent excision (+SLL/CLL)
19	FNA-P	LN	SLL/CLL and mycobacteria	None
20	USG-FNA	LN	SLL/CLL	Surgical biopsy (+SLL/CLL)
21	FNA-P	LN	SLL/CLL	FNA (see below)
22	USG-FNA	LN	SLL/CLL	Surgical biopsy (+SLL/CLL)
23	TP	Lung mass	SLL/CLL and adenocarcinoma	Concurrent surgical excision (+SLL/CLL and adenocarcinoma)
24	TP	LN	SLL/CLL and Ig-containing cells	Concurrent surgical excision (+SLL/CLL and Ig-containing cells)
25	FNA-P	Salivary gland	SLL/CLL	None
26	EBUS-FNA	LN	SLL/CLL and SqCC	None
27	TP	LN	SLL/CLL and seminoma	Concurrent surgical excision (+SLL/CLL and anaplastic seminoma)
28	TP	LN	SLL/CLL	Concurrent surgical excision (+SLL/CLL)
29	TP	LN	SLL/CLL, suspicious for transformation	Concurrent surgical excision (+SLL/CLL with atypical features and increased large cells)

FNA-P, FNA by pathologist; EBUS, endobronchial ultrasound-guided FNA; TP, touch preparation; BM, bone marrow biopsy; PB, peripheral blood flow cytometry; SqCC, squamous cell carcinoma; Ig, immunoglobulin; LN, Lymph node

### Ancillary studies and follow-up

At least one ancillary study was utilized in 28 of 29 cases (97%), including all of the cases with coincident processes. FC was performed in 23 cases (79%), FISH studies in seven cases (24%), special stains (AFB and Grocott stains) 7in two cases (7%) and IHC in 16 cases (55%). The IHC was largely performed for further characterization of the lymphoid cells, including a cyclin D1 stain to exclude mantle cell lymphoma or CD15/LeuM1 and CD30 to exclude HL (14 cases, 88% of the cases with IHC), for further characterization of an epithelial malignancy (one case, 6%) or for both indications (one case, 6%). In one case, polymerase chain reaction (PCR) was performed on the cell block to subtype the mycobacteria identified with the AFB stain. Twenty-one cases (72%) had pathological follow-up (histologic, cytologic or flow cytometry specimens) available in our records, including 15 (71%) with histologic follow-up, four (19%) with cytologic follow-up only and two (10%) with peripheral blood follow-up only.

### Coincident lesions

**Figure 1:**
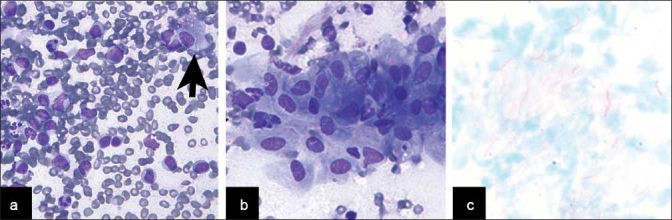
Mycobacterial infection involving a lymph node involved with SLL/CLL. A&B. In a background of lymphoma, the FNA showed histocytes (a. arrow) and granulomas b) with the negative image of mycobacteria on DQ stained smears (A. DQ, ×400; B. DQ, ×600). c) An AFB stain performed was positive for acid fast bacilli (AFB, ×400)

**Figure 2:**
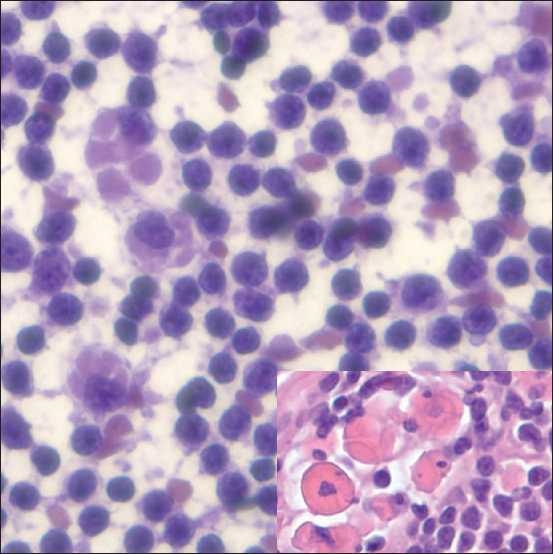
Immunoglobulin-rich histiocytic cells (crystal storage histiocytosis) in a lymph node involved with SLL/CLL. The touch prep of a mediastinal LN demonstrated histiocytic cells with abundant cytoplasm containing dense material and cracking in a background of monomorphic lymphocytes. The corresponding histology (inset) of the LN showed SLL/CLL with similar cells containing immunoglobulin, as confirmed by immunohistochemical stains (TP, hematoxylin and eosin [H&E], ×400; inset, histology, H&E, ×400)

**Figure 3:**
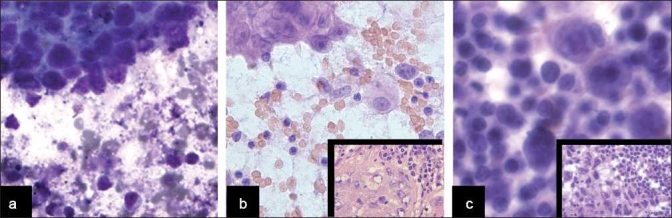
Non-lymphoid malignancies with SLL/CLL. a) Squamous cell carcinoma with SLL/CLL in a LN (fine needle aspiration, Diff-Quik™ [DQ], ×600). b) Adenocarcinoma of the lung with parenchymal lung involvement by SLL/CLL (touch prep [TP], hematoxylin and eosin [H&E], ×400; inset, corresponding histology, H&E, ×400), c) anaplastic seminoma with SLL/CLL in a mediastinal LN (TP, DQ, ×400; inset, corresponding histology, H&E, ×400)

**Figure 4:**
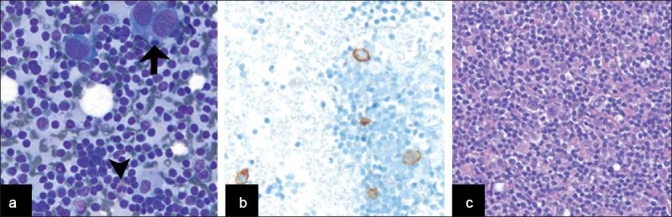
Hodgkin lymphoma arising in a background of SLL/CLL. a) The LN demonstrated several Reed-Sternberg cells (arrow) in a background of a small homogeneous lymphoid population with scattered eosinophils (arrowhead) (Diff-Quik™ [DQ], ×400). b) Immunostains confirmed the impression of Hodgkin’s lymphoma (CD30 immunostain, X200) and FC confirmed the presence of SLL/CLL. c) Histology of LN involved by HL and SLL/CLL (hematoxylin and eosin, X200)

Coincident entities were identified in nine of 29 cases (31%; Table 3) and included non-neoplastic conditions, including infection (one case; Figure 1) and immunoglobulin-containing cells (one case; Figure 2). The neoplastic lesions included squamous cell carcinoma (two cases, Figure 3a), adenocarcinoma (one case; Figure 3b), seminoma (one case; Figure 3c) and HL (three cases; Figure 4).

**Table 3:** Coincident processes identified in 29 cases of SLL/CLL

*Coincident processes with SLL/CLL*	*#cases (%)*	*#cases with follow-up (%)*
Hodgkin lymphoma	3 (10)	1 (33)
Infection (Mycobacteria)	1 (3.5)	0 (0)
Immunoglobulin-containing cells	1 (3.5)	1 (100)
Non-lymphoid malignancies	4 (14)	3 (75)
Adenocarcinoma	1 (3.5)	1 (100)
Squamous cell carcinoma	2 (7)	1 (50)
Anaplastic seminoma	1 (3.5)	1 (100)
Total	9 (31)	5 (56)

In addition, there were six cases with large cells suspicious for transformation, of which five (83%) had clinical follow-up available in our records. On follow-up in these five cases, two cases were diagnosed with HL, two cases had biopsy or excision suspicious for a large cell transformation and one case had a bone marrow biopsy negative for a large cell transformation. Of the three patients with a HL transformation diagnosed by cytology, all were primary diagnoses and one had histological follow-up available in our records confirming the diagnosis.

### Cytomorphologic evaluation

**Figure 5:**
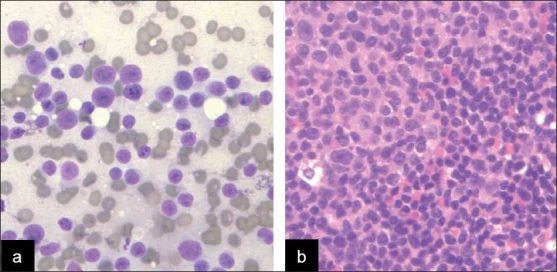
Cytomorphology of SLL/CLL case with large cells suspicious for transformation and histologic follow-up. a) Fine needle aspiration of right axillary LN with large cells suspicious for transformation (Diff-Quik™, × 400). The large cells are approximately 2–2.5× the size of the adjacent red blood cells, with prominent large nucleoli, in a background of the small lymphoid cells characteristic of SLL/CLL. b) The follow-up bone marrow biopsy (hematoxylin and eosin, ×400) was involved by SLL/CLL and also showed large cells with similar features, which were worrisome for an incipient transformation. The peripheral blood also showed an increase in prolymphocytes

All cases showed a relatively homogeneous small lymphoid population with nuclei that range from approximately 1.5 to two-times the size of nearby red blood cells, with clumped chromatin and scant cytoplasm, occasionally seen as eccentrically placed rims of basophilic cytoplasm (imparting a vague plasmacytoid appearance). The presence of carcinoma within the setting of SLL/CLL was clearly evident on cytomorphologic examination due to the presence of large cells with cytologic atypia, including many in cohesive groups, opposed to the small, dyscohesive lymphoid cells [Figure 3a and b]. There was one case of metastatic anaplastic seminoma involving a mediastinal lymph node involved by SLL/CLL, and the seminoma showed dyscohesive cells with large prominent nucleoli without a tigroid background [Figure 3c]. The presence of HL within SLL/CLL was also identified by cytomorphologic examination and identification of characteristic large Reed-Sternberg cells with a CD15/LeuM1- and CD30-positive immunophenotype by immunohistochemistry, a polymorphous background and a neoplastic B-cell population of SLL/CLL [Figure 4]. The case of non-tuberculous mycobacterial infection arising within SLL/CLL showed numerous non-necrotizing granulomas with the negative image of mycobacteria present on high-power examination of the Diff-Quik-stained smears [Figure 1a and b]. An acid fast stain performed on an aspirate smear and cell block section was also positive [Figure 1c] and PCR performed on the cell block confirmed the presence of a non-tuberculous mycobacteria, *Mycobacterium kansasii*. The case of immunoglobulin-rich cells within SLL/CLL showed cells with abundant cytoplasm and round nuclei, which appeared to have somewhat dense cytoplasm and cracking on the touch preparation [Figure 2]. These cells were positive for an immunohistochemical stain for kappa light chain and were morphologically compatible with histiocytes having immunoglobin inclusions, as described in crystal storage histiocytosis. The cases with atypical large cells suspicious for a large cell transformation generally had scattered cells (approximately 10% or more) that were intermediate-to-large in size with prominent nucleoli and a moderate amount of basophilic cytoplasm [Figure 5].

## DISCUSSION

Cytomorphologic assessment of lymph nodes and other palpable lesions is frequently performed to make a primary diagnosis of lymphoma, to evaluate for recurrent disease, to get staging or prognostic information and to evaluate for a transformation to a higher-grade lymphoma. In our review of SLL/CLL cases diagnosed by FNA or intraoperative TPs, simultaneous processes were present in 31% of the cases (nine of 29 cases) and involved a spectrum of lesions ranging from non-neoplastic to neoplastic. Ancillary studies were usd in a majority of the cases (97%) and in all of the cases with coincident lesions. These findings demonstrate the importance of cytomorphologic assessment in patients with SLL/CLL because recognition of a second malignancy may change the prognosis, may alter the treatment and may make the patient ineligible for entry into clinical trials.[5]

In patients with SLL/CLL, population-based tumor registries have reported a variety of other malignancies, including lung cancer, melanoma and large cell lymphomas.[6,30] Our study showed that the most common carcinoma arising with SLL/CLL was squamous cell carcinoma, which has also been reported in the past.[17,19,20] The occurrence of these secondary tumors in SLL/CLL patients may be due to the good prognosis associated with an indolent lymphoma leading to long survival and sporadic occurrence of other tumors, particularly in older patients. Other possible etiologies include genetic predispositions, environmental factors and the potentially immunosuppressive role of the lymphoma itself or the treatment modalities used. The occurrence of non-neoplastic entities have only rarely been reported in association with SLL/CLL, and are predominantly viral infections.[31,32] Our study illustrates two additional non-neoplastic processes seen simultaneously with SLL/CLL, including a case of non-tuberculous mycobacteria [Figure 1] and a case with immunoglobulin-containing histiocytes consistent with crystal storage histiocytosis [Figure 2]. Thus, there is a wide range of neoplastic and non-neoplastic processes to consider when examining cytology specimens in patients with SLL/CLL.

Given that only 0.5–1% of SLL/CLL cases have a variant of RS transforming to a HL,[6,26,27] there are only rare reports of this transformation diagnosed by FNA in the cytology literature.[9,29] Although this is a small percent of cases overall, HL transformations comprise about 15% of Richter transformations and are the second most common coincident hematolymphoid malignancy in these patients.[6] From a clinical standpoint, patients with SLL/CLL and a HL transformation appear to have an aggressive clinical course, but their prognosis is more favorable than that of patients with the typical RS to a large cell NHL.[7,33] Our study found three cases (10%) with HL arising in the background of SLL/CLL, with the diagnosis based on cytomorphology and immunophenotyping of the cells present (by FC for the SLL/CLL and by IHC for the HL). Thus, the occurrence of HL in these patients illustrates the importance of cytomorphologic assessment in SLL/CLL patients and the need for cell block material, because the findings crucial to the diagnosis may not be evident by FC alone and impact the prognosis.

Many papers in the literature have focused on the typical Richter transformation, which is a transformation to a high-grade NHL. In our retrospective review, there was one definitive case of a CD5-positive large cell lymphoma that was diagnosed by FNA, which was confirmed on histologic follow-up and found to be arising in a background of a small cell lymphoma compatible with SLL/CLL. However, this case was excluded from our study given that the FNA did not show a definitive SLL/CLL component. This group of large cell lymphomas is hard to identify in our cytology files, because many will appear as large cell lymphomas and may not show any evidence of the small cell lymphoma that it arose from. In addition, distinguishing a primary diffuse large B-cell lymphoma (DLBCL) from a secondary DLBCL arising from SLL/CLL can be difficult or impossible in FNA material, as well as histological material, particularly because some of these cases will also have a loss of CD5 and CD23.[33,34] Thus, our study does not adequately represent the transformations to a large cell NHL.

Cytomorphologic evaluation in SLL/CLL patients is also important to identify cases with atypical large cells worrisome, but not diagnostic, of a large cell transformation, which may need further clinical follow-up and/or tissue sampling. In our series, there were six cases (21%) suspicious for transformation, of which four (67%) had clinical follow-up in our records. Approximately 50% of the cases suspicious for transformation were confirmed on follow-up, indicating the importance of looking for large cells in patients with SLL/CLL. In the literature, it has been reported that the percent of transformed large lymphocytes that can reliably indicate the presence of a large cell lymphoma ranges from 20% to 40% or greater.[35,36] Our threshold was slightly lower, at 10%, as a more conservative approach to include cases that are not only suspicious for a large cell NHL but also potential cases of CLL with increased prolymphocytes and HL transformations in SLL/CLL. Although large cells may indicate a higher grade lymphoma, there have also been reports of other processes causing atypical large cells, resembling Reed-Sternberg cells, arising within SLL/CLL following fludarabine treatment,[37] and in reactive hyperplasia due to viral infection.[31,32,38] In addition, sampling of proliferation centers in SLL/CLL (pale zones identified on histology) with a high content of prolymphocytic cells or paraimmunoblasts could also theoretically give rise to an increased number of larger cells in aspirates.[2] Thus, in scenarios when there are atypical large cells, we typically recommend obtaining sufficient material for cell block to perform IHC to exclude an epithelial malignancy or HL, and further tissue sampling to exclude the possibility of a large cell transformation, CLL with increased prolymphocytes or other processes.

The risk of a coincident process, including the risk of HL, in our study is slightly higher than that reported in population-based tumor registries and clinical studies, and may be due to a variety of factors.[6,26,27] Given that our institution is a tertiary care referral center with a cancer center and an active hematologic–oncology service following patients with lymphoma, FNA is frequently used to evaluate patients with a history of lymphoma in order to look for a high-grade transformation, as described by other institutions.[9] Thus, we have many patients with a known history of SLL/CLL (79% in this study), who are referred when they become symptomatic or have a change in their clinical course, because FNA is often the fastest and easiest way to get a diagnosis. Another possible explanation is that cytology is being used less often to make the primary diagnosis of SLL/CLL, given that the diagnosis can also be made on peripheral blood or excisional biopsies. Thus, our cases may underrepresent the bulk of SLL/CLL patients with early indolent disease and cause our overall percent of a coincident process to be higher than that seen in population-based studies. Finally, it has been shown that the incidence of transformation increases with the number of previous treatment regimens,[26] and because we are a referral center, we may have more patients undergoing treatment. Overall, these findings suggest that the chance of detecting a coincident process by cytology at a tertiary care center may be higher, given that it is frequently used in patients with a history of lymphoma who may have had a variety of treatment regimens, and in patients with a high clinical suspicion for a transformation or secondary process.

In conclusion, our report characterizes the spectrum of lesions that can occur within a background of SLL/CLL diagnosed by FNA or TP. Cytomorphologic assessment is crucial in determining whether there is a coincident process (such as a large cell transformation, metastatic malignancy or infectious process), as these findings may not be evident by FC evaluation alone and may alter the triage of the case (i.e., acquiring material for cell block in addition to FC), in addition to altering the treatment and prognosis for the patient.

## COMPETING INTEREST STATEMENT BY ALL AUTHORS

No competing interest to declare by any of the authors.

## AUTHORSHIP STATEMENT BY ALL AUTHORS

Each author acknowledges that this final version was read and approved. All authors qualify for authorship as defined by ICMJE http://www.icmje.org/#author Each author participated sufficiently in the work and takes public responsibility for appropriate portions of the content of this article.

## ETHICS STATEMENT BY ALL AUTHORS

This study was conducted with approval from institutional Review Board (IRB) (or its equivalent) of all the institutions associated with this study.

## EDITORIAL / PEER-REVIEW STATEMENT

To ensure integrity and highest quality of CytoJournal publications, the review process of this manuscript was conducted under a double blind model(authors are blinded for reviewers and reviewers are blinded for authors)through automatic online system.
